# Effect of chitosan nanoparticles loaded with platelet lysate on in vitro fertilization and early embryo development in a mouse premature ovarian failure model

**DOI:** 10.22038/ijbms.2025.84330.18249

**Published:** 2025

**Authors:** Fereshte Torabi, Sareh Karimi, Fatemeh Alipour, Mohamad Javad Mirzaei-Parsa, Alireza Ebrahimzadeh-Bideskan

**Affiliations:** 1 Department of Anatomy and Cell Biology, Faculty of Medicine, Mashhad University of Medical Sciences, Mashhad, Iran; 2 Pathology and Stem Cell Research Center, Kerman University of Medical Sciences, Kerman, Iran; 3 Applied Biomedical Research Center, Mashhad University of Medical Sciences, Mashhad, Iran

**Keywords:** Chitosan, Cyclophosphamide, *In vitro* fertilization, Platelet-rich plasma, Premature ovarian failure

## Abstract

**Objective(s):**

Premature ovarian failure (POF) is a common cause of women’s infertility. Cyclophosphamide (CP) is one of the most important gonadotoxic agents that lead to POF. This study aimed to evaluate the potential therapeutic effect of chitosan nanoparticles loaded with platelet lysate (PLCH NPs) on *in vitro *fertilization (IVF) outcome and early embryo development in a model of CP-induced POF.

**Materials and Methods:**

In this study, synthesized nanoparticles were developed based on the ionic gelation method and characterized by the physicochemical properties of particle size, zeta potential, FTIR, microscopic studies, drug encapsulation, and *in vitro *drug release. Sixty female BALB/c mice were randomly assigned into six groups: Control, sham, POF, POF-PL, POF-CH, and POF-PLCH. Finally, the PLCH NPs were examined for their therapeutic potential against CP-induced POF by evaluating Anti-Müllerian hormone (AMH) levels, malondialdehyde (MDA) levels, total anti-oxidant capacity (TAC), fertilization rate, and embryo development.

**Results:**

The results showed that PL’s *in vitro* release profile has a sustained release pattern. The treatment of PLCH NPs in POF mice increased AMH and TAC and decreased MDA levels compared to the control group (*P*<0.05). The mean number of retrieved oocytes, cleavage and fertilization rates, and blastocyst formation rates were significantly increased in the POF-PLCH group compared with the POF group (*P*<0.05).

**Conclusion:**

This study proposes a novel PLCH NP-mediated combination therapy for the clinical treatment of POF and shows that PLCH NPs are superior to free PL in terms of effectiveness.

## Introduction

Infertility is determined as the inability to conceive after 12 months of sexual activity without protection and affects 15–17% of couples worldwide. The World Health Organization has recognized infertility as a global public health topic (1, 2), and about 50% of infertility cases are related to female factors. The most common reasons for female infertility are premature ovarian failure (POF), polycystic ovarian syndrome (PCOS), uterine factors, including thin endometrium and intrauterine adhesions, and menstrual and ovulation disorders (3). 

POF is one of the common causes of infertility in women at the age of 40 and is defined by amenorrhea, low estrogen levels, high gonadotropin levels, ovarian atrophy, lack of mature follicles, and infertility. In recent years, the prevalence of POF has increased from 1% to 2% among women of fertility age (4, 5). Although the causes of POF are highly heterogeneous, iatrogenic causes (radiotherapy and chemotherapy) are responsible for most of the cases. One of the common medications that is mainly used in the treatment of malignancies is Cyclophosphamide (CP). Despite its broad-spectrum clinical uses, CP exhibits potent gonadotoxicity, hence increasing the risk of ovarian failure in patients (6). Although assisted reproductive technology (ART), including intrauterine insemination (IUI), *in vitro* fertilization (IVF), and intracytoplasmic sperm injection (ICSI) are excellent options for the chance of conceiving a child in cancer survivors who have infertility, sometimes patients need more treatments options to improve ovarian function after chemotherapy and subsequent POF (7, 8).

Ovarian rejuvenation is a method to maintain ovarian function and improve ovarian response to activation (9). Platelet-rich plasma (PRP) is one of the proposed therapeutic strategies used in regenerative medicine, specifically in reproductive medicine, and has been used since around 5 years ago (10, 11). PRP contains a plethora of cytokines and growth factors (GFs) for tissue regeneration (PDGF, TGF-β1, VEGF, bFGF, IGF-1, EGF, and HGF) (2, 12, 13). The advantage of PRP is its autologous nature in humans, so there is no risk of immune and allergic reactions and transmission of microorganisms. Furthermore, because of its convenient preparation, low cost, antimicrobial effect, and easy accessibility, as well as its being a non-invasive therapeutic intervention, PRP has become an area of ​​interest in reproductive medicine (11, 14, 15). Despite the benefits of PRP, its clinical application is limited due to the short half-life of its GFs, limited mechanical strength, rapid degradation by lytic enzymes, and short-term release of bioactive GFs (16). On the other hand, Platelet lysate (PL) is also considered an equal and safe source of autologous GFs, presenting the advantage of storage and delivery at particular time points, limiting the long time needed for PRP preparation (17). 

Two hypotheses have been proposed regarding how PRP can improve oocyte retrieval in women with ovarian failure: GFs in PRP can awaken dormant ovarian oocytes, and PRP provides suitable conditions for the differentiation and development of uncommitted ovarian stem cells into new oocytes (2, 12, 18). Numerous studies have demonstrated that PRP improved the IVF outcomes in poor prognostic infertile women, resulting in live births (14, 17–20). Therefore, PRP appears suitable for treating ovarian reserve loss because it improves the ovarian microenvironment and promotes ovarian angiogenesis, folliculogenesis, and hormonal profile after intraovarian injection of PRP (13, 21–23).

Chitosan (CH) is a natural, biocompatible, biodegradable, non-toxic, and inexpensive polysaccharide with a high loading capacity that has been FDA-approved for use in biomedical fields (24–26). It also possesses anti-oxidant, antimicrobial, antitumor, radical removal, and anti-inflammatory properties. Moreover, CH can adhere to mucosal surfaces, increase penetration, and slow down and control the release of bioactive components (27–29). One of the possible ways to overcome the problems of PL release is to encapsulate it in a CH polymer to release it sustainably (14).

Therefore, the present study aimed to investigate the effectiveness of PL and CH in a sustained release formulation on *in vitro* fertilization rate and early embryo development in the mouse ovarian failure model induced by CP.

## Materials and Methods

### Preparation of platelet-rich plasma and platelet lysate

**Figure 1:**
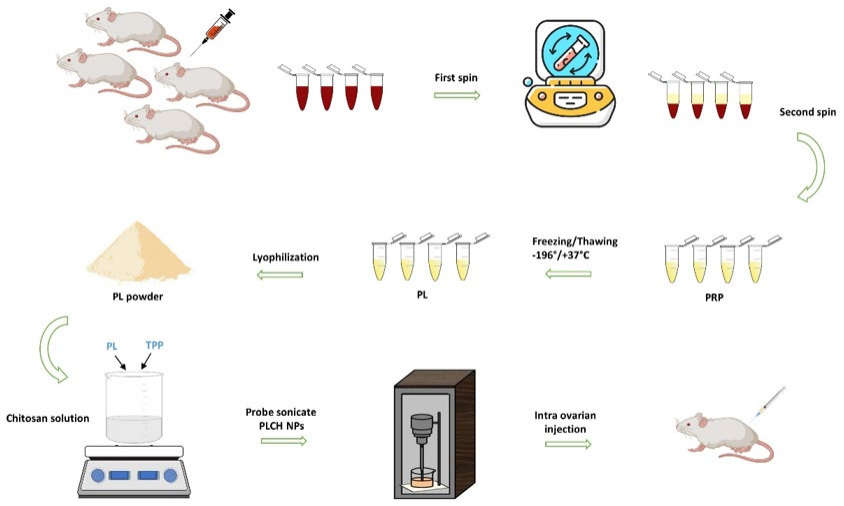
Schematic illustration of PLCH NPs synthesis process. The process commenced with isolating PRP from the blood of BALB/c mice. Subsequently, PL is obtained through a freeze-thaw process. The formation of the cloudy solution upon the addition of PL and TPP to the chitosan solution confirmed the successful synthesis of nanoparticles. These nanoparticles are then subjected to sonication and freeze-drying

PRP: Platelet-rich plasma; PL: Platelet lysate; TPP: Tripolyphosphate; PLCH NPs: Platelet lysate-loaded chitosan nanoparticles

Ten adult female BALB/c mice (8–10 weeks old, 24–30 g) were used to prepare PRP. The mice were deeply anesthetized (ketamine 100 mg/kg + xylazine 10 mg/kg), and whole blood was collected directly from the heart apex. The blood samples were then transferred into vacuum tubes containing 3.2% sodium citrate (A191237B, Greiner Bio-One, Australia). PRP was prepared using a two-step centrifugation process. After the first centrifugation at 1200 rpm for 20 min, the supernatant was transferred to another tube and centrifuged at 2500 rpm for 15 min (30). The top layer (upper ⅔) consisting of platelet-poor plasma (PPP) was removed, and the bottom of the tube was stored as PRP. Extract PRP was around 5 times higher than the blood platelet count. Finally, the PRP was aliquoted and frozen at -20 °C for further experiments. To obtain PL, PRP samples were exposed to multiple freezing (-196 °C for one minute) and thawing cycles (37 °C for six minutes) to ensure platelet lysis. After thawing, PL was centrifuged at 4 °C, 9000 rpm for three minutes to remove cell debris. Finally, the samples were freeze-dried for 24 hr (Figure 1) (30). 

### Preparation of chitosan nanoparticles (CH NPs)

Chitosan (low molecular weight, Sigma) solution (0.2 %) was prepared by adding chitosan powder to 1% acetic acid incrementally under continuous magnetic stirring. The pH of the solution was adjusted to 5.5 ± 0.2 using 0.1 M sodium hydroxide solution. Next, tripolyphosphate solution (TPP) at concentration 0.25 % (w/v) was added dropwise to the CH solution at a 1:5 ratio, followed by incubation with stirring in a magnetic stirrer at 600 rpm for 60 min. The prepared solution was then sonicated with a probe-type sonicator (Bandelin Sonoplus UW 3200, Berlin, Germany) in an ice bath at 80w power output under a pulsed operation mode (80% on/ 20% off) for ten minutes. The formation of a cloudy solution after stirring confirmed the presence of nanoparticles. The final prepared solution was stored and sealed at 4 °C.

### Preparation of PL-loaded chitosan nanoparticles (PLCH NPs)

PLCH NPs in this study were prepared using the ionic gelation method. Briefly, 1 ml of 0.25 % (w/v) TPP was added dropwise to 5 ml of CH solution (0.2 % (w/v)) containing 50 mg of PL under constant stirring at 25 °C. Then, the prepared solution was sonicated, similar to the synthesis CH NPs method (Figure 1).

### Characterization of nanoparticles

The hydrodynamic size and surface ζ potential (ZP) of nanoparticles were measured using dynamic light scattering (DLS, Cordouan Technologies) and Zetasizer Nano (SZ-100 HORIBA Scientific, Japan), respectively. 

Field emission scanning electron microscopy (FESEM, MIRA3 TESCAN, Czech Republic) was used to observe the morphology of the nanoparticles. For this analysis, the samples were diluted 50 times using double distilled water and dropped on an aluminum stub. The air-dried stub was placed in an automatic fine gold coater at 10 mA for 90 sec. The PLCH NPs were observed at 10 kV acceleration voltages and imaged. The chemical bonds and chemical groups of the PLCH NPs were evaluated by FTIR spectroscopy (PerkinElmer- Norwalk, USA). The scanning wave range was 400 to 4000 cm^-1^ with a resolution of 4 cm^-1^ at ambient temperature. KBr was used to mix the dried samples (1 mg) and press them on a hydraulic press at 20 °C for spectra analysis. 

To measure the encapsulation efficiency (EE %), the un-entrapped PL was separated from PLCH NPs by centrifuging at 13,000 rpm for 20 min. The absorbance of free PL in the supernatant was measured using a UV spectrophotometer at 280 nm. The EE% was calculated using the following relation:

EE % = (Total amount of PL - Free PL) / Total amount of PL × 100

The *in vitro* release of PLCH NPs was performed using a cellulose dialysis bag (cut off 12 kDa). Briefly, 2 ml of PLCH NPs were placed in dialysis bags, immersed in 25 ml of phosphate-buffered saline (PBS) at 37 °C, and stirred magnetically at 100 rpm. At predetermined intervals (1, 6, 12, 24, and 48 hr), 2 ml of release medium was withdrawn and replaced with fresh PBS. The samples were analyzed using a UV-visible spectrophotometer at 280 nm to determine the cumulative drug release over time.

### In vivo study design

Sixty Female BALB/c mice (8–10 weeks old, 24–30 g) were obtained from the animal house of the Faculty of Medicine, Mashhad University of Medical Sciences, Mashhad, Iran. The animals were kept in a standard condition (temperature 23 ± 2 °C, humidity 50 ± 10, 12 hr /12 hr light/dark cycle) with free access to food and water. Additionally, ten female BALB/c mice were obtained for PRP preparation and 12 adult male BALB/c mice for IVF. All procedures were reviewed and approved by the Ethics Committee of Mashhad University of Medical Sciences, Mashhad, Iran (with approval No. IR.MUMS.MEDICAL.REC.1402.077). Also, all institutional and national guidelines, as well as ethical considerations, were followed according to the National Institutes of Health Guide for the Care and Use of Laboratory Animals (NIH Publications No. 80-23, revised 1978).

After a week of acclimation, the animals were divided into six groups (n=10): Control (without any intervention), sham (intra-ovarian injection of normal saline), POF, POF-PL, POF-CH, and POF-PLCH. The POF model was established via a single intraperitoneal injection of CP (150 mg/kg) (31). The POF model establishment was validated by assessing the ovarian histological examination. 

### Intra-ovarian injection

For the intra-ovarian injection, the animals were anesthetized with IP injection of 70 mg/kg ketamine and 10 mg/kg xylazine. Seven days after the CP administration and intra-ovarian injection, bilateral dorsolateral skin incisions were made in the supra-flank position using aseptic surgical procedures (70% ethanol and 10% iodopovidone solutions). Subsequently, a small incision was made on both sides to access the right and left ovaries: 10 µl of PL, CH NPs, PLCH NPs, and normal saline were injected into each ovary. Ultimately, the skin was sutured and disinfected with an iodopovidone solution. After surgery, mice were administered acetaminophen (1 mg/ml drinking water) and 50 mg/kg of Neomycin to prevent bacterial infections for three days (32).

### Ovarian stimulation 

At the end of the treatment period, ovarian stimulation in all experimental groups was carried out by intraperitoneal injection of 10 IU pregnant mare serum gonadotropin (PMSG, GONASER; SPAIN) followed by the administration of 10 IU human chorionic gonadotropin (HCG, PD PREG) 48 hr later. Animals were sacrificed 14–15 hr after the HCG injection and fallopian tubes were removed to extract the cumulus-oocyte complexes (COC) under a stereomicroscope (33).

### Sperm collection

Before the oocyte collection, the study used 12 mature male BALB/c mice (8–10 weeks old, 26–30 g) for IVF processes. After being deeply anesthetized intraperitoneally with 100 mg/kg ketamine and 10 mg/kg xylazine, the animals were sacrificed by cervical dislocation, and immediately, the epididymis was removed and dissected in warm Hams F10 medium (Biowest, Cat. n: P0146-N10L) with 10% albumin. After washing with a centrifuge (1500 rpm, 4 min), the supernatant was removed, and the plate was incubated with 0.5 CC Hams F10 medium with 10% albumin at 37 °C, 5% CO_2_. After 45 min, the swim-up sperm was used for IVF.

### Oocyte quality and collection, in vitro fertilization (IVF), and embryo development evaluations

The following morphological criteria were employed to evaluate oocyte quality. 1) normal oocytes and 2) abnormal oocytes (granular or dark cytoplasm, thick or abnormal zona pellucida (ZP), cytoplasmic fragments, large perivitelline space, and abnormalities in oocyte shape) (34). 

The COCs were collected in droplets of IVF medium under liquid mineral oil. Then, a suitable sperm count (1 × 10^6^ sperm/ml) was added to each drop. Five hours after sperm addition, the fertilization rate was evaluated under a stereomicroscope by checking pronuclei (2PN) in zygotes.

Afterward, the zygotes were washed and transferred into a fresh culture medium (ORIGIO Sequential Series, Denmark) that had already reached the equilibrium and cultured (37.5 °C in 5% CO_2_). After 72 hr, the culture medium was changed with Blast medium (ORIGIO Sequential Series, Denmark) (37.5 °C in 5% CO_2_). The embryo development at the 8-cell (cleavage) and blastocyst stages were evaluated 72 and 120 hr after IVF. 

### Biochemical analysis

At the end of the study, mice from each group were anesthetized, and the blood samples were obtained transcardially, followed by centrifugation at 3200 rpm for 15 min. The obtained serum was then stored at -70 °C for biochemical and hormonal analyses. Malondialdehyde (MDA, Navand Lab Kit, Iran), total anti-oxidant capacity (TAC, Kushanzist, KZTA-OB, Iran), and anti-Mullerian hormone (AMH, LDN, LOT 230364A, Germany) were measured using commercial ELISA kits according to the manufacturer’s instructions.

### Statistical analysis

All data were analyzed using GraphPad Prism (v 8.0). Normal distribution of data was tested using the Kolmogorov–Smirnov test. Differences between groups were evaluated by one-way ANOVA and *post hoc* Tukey’s test. Data are expressed as the means ± SEM. *P*<0.05 were considered statistically significant. 

## Results

### Characterization of PLCH NPs

**Figure 2:**
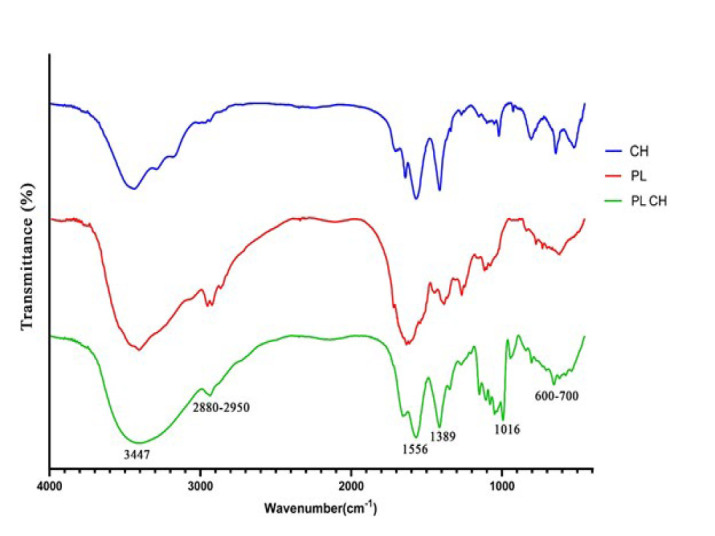
**. **(A) Morphological characteristics of platelet lysate-loaded chitosan nanoparticles (PLCH NPs) using scanning electron microscopy. As shown in the Figures, the FESEM analysis confirmed the presence of consistent structures and the spherical morphology of shape at different scale bars (Arrow). (B) The surface ζ potential (ZP) of PLCH NPs was measured using Zetasizer Nano. (C) The hydrodynamic size of PLCH NPs was measured using dynamic light scattering

The zeta potential and mean hydrodynamic diameter of nanoparticles were obtained at 17 mV and 373±0.4 nm, respectively (Figure 2 B-C). The zeta potential is critical in understanding the surface charge characteristics of NPs. A higher zeta potential results in greater electrostatic repulsion forces between the particles, which leads to greater particle separation, reducing the extent of aggregation caused by Van de Waals interactions (35). The morphology of nanoparticles was analyzed by FESEM and showed consistent and spherical structure at different scale bars (Figure 2 A).

**Figure 3:**
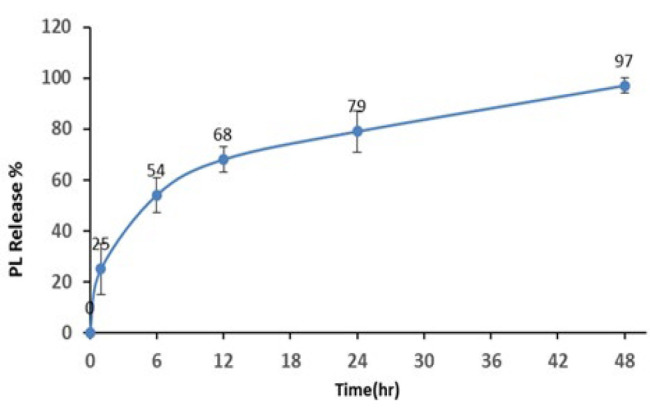
FTIR spectra of CH, PL, and PLCH NPs

The FTIR spectral investigation for PL, CH, and PLCH NPs is shown in Figure 3. FTIR spectra for samples of pure CH and PL powders were used as references to native peaks. The interactions of CH with PL caused a slight shift in chitosan peaks. Regarding Figure 2, the observed broad absorption peak at 3347 cm ^-1^ may be related to a hydroxyl group (O–H) stretching. In addition, the absorption bands at 2880-2950, 1556, 1389, 1016, and 600-700 cm ^-1^ are attributed to C–H, N–H, C–CH3, C–O–C, and C–H and N–H groups, respectively (30, 36, 37). These results confirmed the integration of PL and CH in the prepared NPs.

Encapsulation efficiency (EE) was measured to observe the amount of PL encapsulated in the NPs. Our results showed that the encapsulation efficiency of the PLCH NPs was 71±3%.

**Figure 4:**
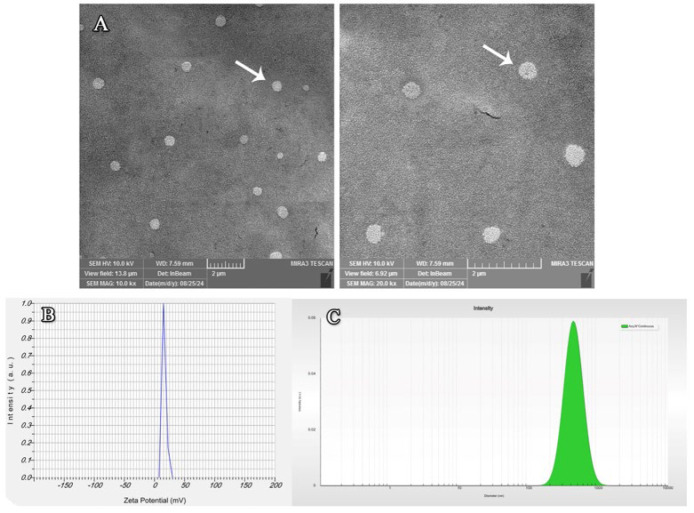
Sustained release pattern of PL from the PLCH NPs was performed using a cellulose dialysis bag at intervals 1, 6, 12, 24, and 48 hr

The PL-release behaviors of NPs were measured using a cellulose dialysis bag at pH 7.4 and 37 °C. As seen in Figure 4, 50% of the PL was released in the first six hours, but the remainder was released within 48 hr and reached 97%. These results showed that PLCH NPs could maintain a sustained release of PL for a relatively long time.

### Establishment of the POF model

**Figure 5:**
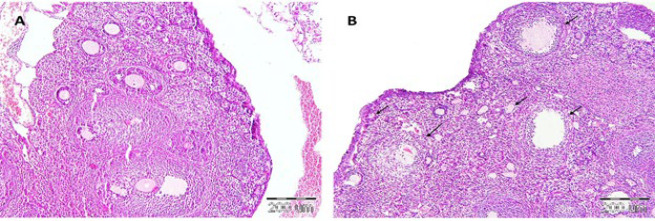
Confirmation of POF modeling using H&E staining

Histopathological evaluations in the healthy control group revealed a normal ovarian tissue structure and morphology. Following CP administration for POF induction, degeneration of granulosa cells and follicular atresia were observed at all stages of follicular development, including primary, secondary, and antral follicles. Accordingly, more atretic follicles were observed in the POF mice than in the control mice. These data confirmed that CP can induce pathological changes in the ovarian stroma, leading to atresia and follicular depletion (Figure 5).

### IVF outcome

**Figure 6:**
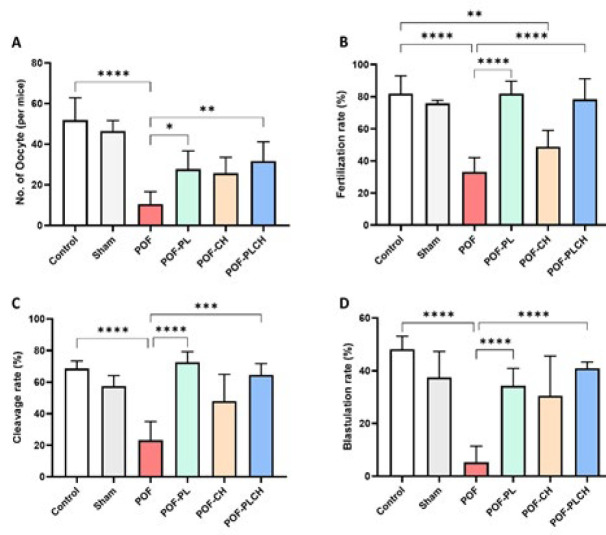
Number of oocytes and early embryo development following different treatments in the studied groups

**Figure 7:**
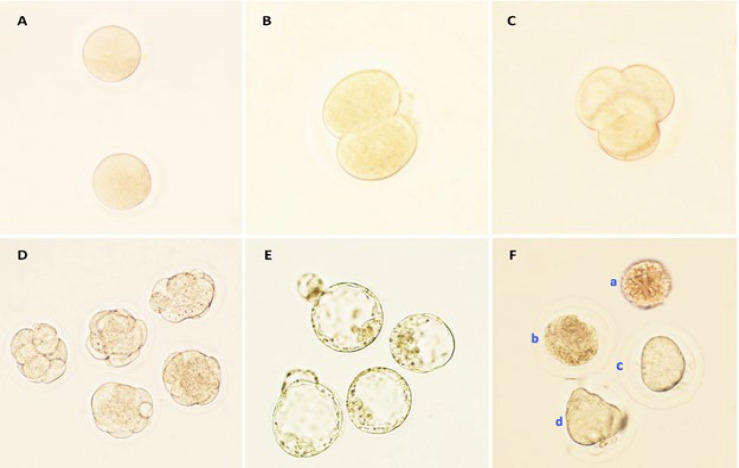
A-E) Photomicrographs show the timeline of preimplantation development of normal oocytes (× 200). (A) Oocyte; (B (Two-cell embryo; (C (Four-cell embryo; (D (Morula; (E (Blastocyst; (F (Types of abnormal oocytes (a: oocyte without a zona pellucida (ZP), b: degenerated oocyte, c: oocyte with large perivitelline space (PVS) and d: dysmorphic oocyte and fragmented polar body)

According to Figure 6, the number of retrieved oocytes, fertilization rate, cleavage rate, and blastocyst formation were significantly decreased in the POF group compared with the control group (*P*<0.05). Moreover, the POF group had a higher proportion of abnormal oocytes than the control group (data not shown). The number of oocytes per mouse was significantly decreased in the POF group compared with the control group (*P*<0.0001). However, the highest number of retrieved oocytes was seen in the POF-PLCH group compared to the POF group (*P*<0.05) (Figure 6 A). The fertilization rate was significantly decreased in the POF (*P*<0.0001) and POF-CH groups (*P*<0.05) compared to the control group. The fertilization rate was significantly increased in the POF-PL and POF-PLCH groups compared to the POF group so the highest percentage of fertilization was observed in the POF-PL group after the control group (*P*<0.0001) (Figure 6 B). The cleavage rate was also significantly decreased in the POF group compared to the control group (*P*<0.0001). However, the rate of cleavage was increased in other treated groups compared to the POF group, so the highest percentage of cleavage was seen in the POF-PL group (*P*<0.0001) (Figure 6 C). The blastocyst formation rate was significantly decreased in the POF group compared to the control group (*P*<0.0001). However, blastocyst formation was increased in all treatment groups compared to the POF group, so the highest rate of blastocyst formation was observed in the POF-PLCH group (*P*<0.0001) (Figure 6 D). The timeline microscopic imaging of the normal development of oocyte into blastocyst is shown in Figure 7.

### AMH level

**Figure 8:**
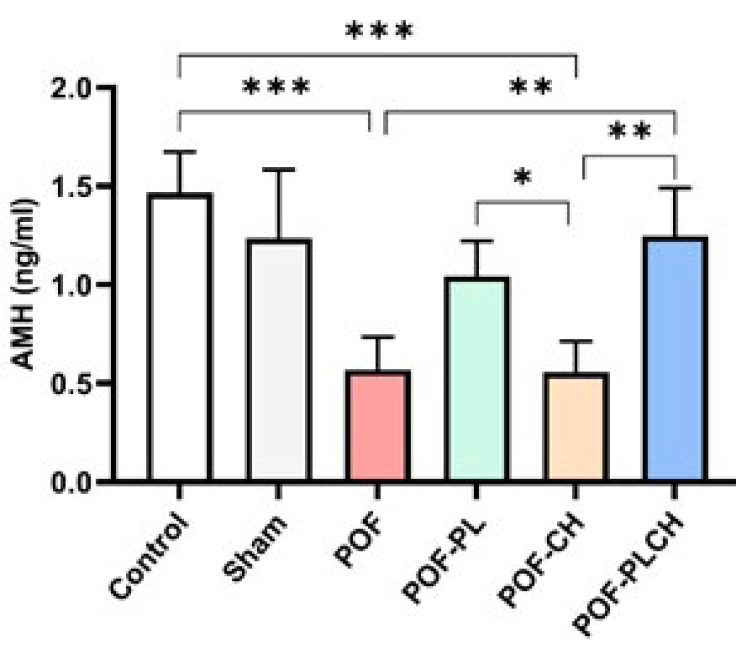
Serum AMH level after treatment in different studied groups, n=4

As shown in Figure 8, the AMH serum level was significantly decreased in the POF and POF-CH groups compared to the control group (*P*<0.001); however, the PLCH NPs resulted in a high concentration of AMH close to the control (*P*<0.01). Although rats treated with CH and PL alone could increase AMH levels compared to the POF group, this difference was not significant. 

### Biochemical assessments

**Figure 9:**
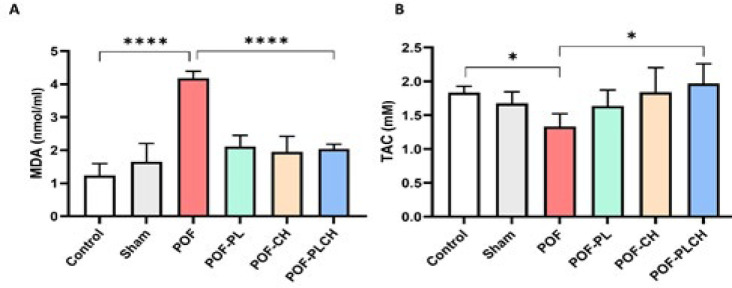
MDA concentration and TAC in the serum of studied groups, n=4.

Serum MDA level was markedly higher in the POF group than in the control group (*P*<0.0001). Moreover, a significant reduction in the MDA level was observed in the POF-PL, POF-CH, and POF-PLCH groups in comparison with the POF group (*P*<0.0001) (Figure 9 A).

Results also revealed that the TAC level was significantly decreased in the POF group compared to the control group (*P*<0.05). However, the TAC level in the POF-PLCH group had the highest level among other POF groups (*P*<0.05) (Figure 9 B).

## Discussion

POF is the most important side effect of chemotherapy that significantly affect the life quality of young women (38). The present study investigated the potential overprotective effects of PLCH NPs on IVF outcomes, such as oocyte quality and early embryo development in the POF model. Alkylating metabolites in chemotherapy drugs have strong gonadotoxic activity, resulting in the loss of the primordial follicle pool (39). Ovarian damage due to CP is ovarian microvascular damage, maldevelopment of follicles, and oxidative stress that leads to oocyte degeneration, granulosa cell apoptosis, and sexual hormone disruption (40).

 PRP is a blood plasma fraction with a platelet concentration 4–5 times higher than the normal level. Due to its tissue regeneration, angiogenesis activation, and anti-inflammatory properties, it has been used in many therapeutic fields (14). PL is a type of PRP obtained by lysing the platelet through the freezing and thawing process; contains all the vital GFs secreted by platelets, including PDGFs, VEGF, IGF, FGF, and TGF-b, and is widely applied for managing soft tissue damage (30, 41). Lyophilized PL was first produced by Keib in 2017. Based on growth factors testing of PRP derivatives using different techniques, the highest concentration of GFs has been reported in lyophilized PRP powder (16). In addition, removing platelet debris via centrifugation makes PL a suitable alternative to PRP due to lower immunogenicity (30).

In our study, PLCH NPs were fabricated using the dialysis method and characterized for physicochemical properties. The encapsulation efficiency showed that PL was well encapsulated in CH NPs, and the loading capacity was more than 70%, which showed that the encapsulation method was well established. FESEM analysis showed spherical NPs in shape, and the particle size distribution using DLS confirmed an average size of 373 nm. Also, the potential of PLCHNPs was studied as a slow-release system. Based on the diagram, the release profile showed that PLCH NPs can maintain PL release for several hours, while free PL quickly completed its release. As mentioned earlier, the development of PLCH NPs had been intended to investigate the prolongation of the release of GFs. In the present study, PLCH NPs were injected into the cortex of both ovaries of POF mice. Chitosan, one of the most widely used biomaterials, has found vast applications in research for various illnesses due to its excellent biocompatibility and biodegradability properties (42). Intra-ovarian injection not only does not reduce the number of NPs but also prolongs their residence time. It also prevents the disadvantages of other injection methods, such as pulmonary embolism caused by tail vein injection, loss of particles caused by intraperitoneal injection, and the short life of the particles. Therefore, the PLCH NPs will have long-term therapeutic effects on the damaged ovarian tissue in this method. Previous studies have shown that CP affects the oocytes more than the granulosa cells (43). In the POF group, the number of retrieved oocytes, cleavage rate, fertilization rate, and blastocyst formation significantly decreased, and the rate of abnormal oocytes increased compared to that of the control group. Consistent with our results, the other study showed that CP injection significantly decreased the average number of retrieved oocytes and the fertilization rate, but contrary to our findings, the rate of morula and blastocyst formation remained the same as the control group (44). In another study on poor ovarian reserve mice, it was observed that PRP does not affect the MII oocyte yield but could improve the number of obtained 2-cell embryos, fertilization rate, formation, and quality of blastocysts. Finally, they concluded that this positive effect was probably due to the increase of local paracrine signaling through the release of GFs in PRP-treated ovaries (45). In the present study, the POF-PL and POF-PLCH groups significantly improved the abovementioned parameters. However, PLCH NPs were more effective in maintaining ovarian function and IVF outcomes following POF.

AMH is a member of the TGF-β superfamily and plays an important role in the recruitment of ovarian follicles. AMH is mainly secreted by the granulosa cells of preantral and small antral follicles. It is a negative regulator of primordial follicle recruitment and subsequent transition into primary follicles (39, 46). It has been reported that the serum AMH level indicates the growing follicles’ population and reflects the ovarian follicular reserve (47). Measuring AMH levels is a sensitive indicator of ovarian function after chemotherapy (48). In our study, CP significantly decreased serum AMH levels, and previous studies confirmed our results (46–48). Also, Luan *et al*. (2019) showed that CP injection reduced AMH levels, then subsequently increased compared to the baseline. The authors suggested that the increase in AMH levels is due to the rise in the number of growing early-stage follicles due to the over-recruitment of dormant primordial follicles (31). In another study, intraovarian injection of PL in women with POI showed promising results in pregnancy with increased AMH levels (17). In the present study, PLCH NPs effectively increased serum AMH levels compared to the POF group. In other words, these NPs may prevent CP-induced ovarian damage by increasing the AMH level.

On the other hand, CP administration induced oxidative stress by increasing MDA levels and decreasing TAC levels, while injection of PLCH NPs improved reproductive performance by increasing total anti-oxidant capacity and decreasing MDA levels. MDA, one of the products of lipid peroxidation, quickly mixes with biomolecules and disrupts glucose metabolism. Studies have shown that oxidative stress leads to ovarian failure by inhibiting oocyte maturation, inducing apoptosis, and damaging embryonic development (49, 50).

The scanning wave range was 400 to 4000 cm^-1^ with a resolution of 4 cm^-1^ at ambient temperature. The interactions of CH with PL caused a slight shift in chitosan peaks. CH: Chitosan; PL: Platelet lysate; PLCH NPs: Platelet lysate loaded chitosan nanoparticles

According to the figure, PLCH NPs could maintain a sustained release of PL for a relatively long time. PL: Platelet lysate; PLCH NPs: Platelet lysate-loaded chitosan nanoparticles

Photomicrograph of the control group (A) showed a normal ovarian tissue structure and morphology. In contrast, degeneration and follicular atresia (arrow) were observed in the CP-treated group (B). POF: Premature ovarian failure; CP: Cyclophosphamide

(A) Total number of oocytes in each group. (B) Fertilization rate in each group. (C) Cleavage rate in each group. (D) Blastulation rate in each group, n= 6. Data represent the means ± SM. *P<0.05, **P<0.01, *** *P*<0.001, and **** *P*<0.000. POF: Premature ovarian failure; POF-PL: Premature ovarian failure plus platelet lysate; POF-CH: Premature ovarian failure plus chitosan; POF-PLCH: Premature ovarian failure plus platelet lysate and chitosan

Data represent the means ± SM. **P<0.01, ***P<0.001. POF: Premature ovarian failure; POF-PL: Premature ovarian failure plus platelet lysate; POF-CH: Premature ovarian failure plus chitosan; POF-PLCH: Premature ovarian failure plus platelet lysate and chitosan

Data represent the means ± SM. * *P*<0.05, **** *P*<0.0001. POF: Premature ovarian failure; POF-PL: Premature ovarian failure plus platelet lysate; POF-CH: Premature ovarian failure plus chitosan; POF-PLCH: Premature ovarian failure plus platelet lysate and chitosan; MDA: Malondialdehyde

### Suggestion/Limitation

According to our results, PLCH NPs might be beneficial for infertility clinics. However, further research with larger sample sizes, longer-term studies, and clinical trials is needed to assess the safety and effectiveness of PLCH NPs comprehensively. In addition, some limitations are noted. Considering the adverse effects of CP on fertilization, blastocyst apoptosis, intraoocyte spindle integrity, and evaluation of Zp1, Zp2, Zp3, and Nlrp5 genes involved in fertilization were not investigated. Nevertheless, this study forms a relevant justification for future investigations and suggests a promising potential therapeutic agent for ovarian rejuvenation of cancer survivors.

## Conclusion

Our study showed that intraovarian injection of PLCH NPs could restore ovarian function against CP-induced damage and improve the number of oocytes and developing embryos. Thus, this study suggests a new therapeutic strategy combining PL and CH for the clinical treatment of POF versus using PL therapy alone.

## Declaration

We have not used any AI tools or technologies to prepare this manuscript.
